# Supplementary material for: Manganese exposure in juvenile C57BL/6 mice increases glial inflammatory responses in the substantia nigra following infection with H1N1 influenza virus
Source: PLoS One. 2021 Jan 25;16(1):e0245171. doi: 10.1371/journal.pone.0245171 (PMC7833173; doi:10.1371/journal.pone.0245171)
Supplement: Attachment — Submitted filename: PONE-D-20-34080_R1_response to reviews.pdf [file pone.0245171.s003.pdf]

**PONE-D-20-34080-R1**

**Response to Reviewer Concerns**

1. “Please ensure that your manuscript meets PLOS ONE's style requirements, including those for file naming.”

The manuscript has been carefully revised to insurance compliance with the style requirements for PLoS ONE, including file naming.

2. “In your Methods section, please provide additional details regarding the animals used in your study and ensure you have described the source.”

The Methods have been updated in the revised manuscript (p. 5, paragraph 1) to note the source vendor of the animals to add details on husbandry.

3. “We note that you are reporting an analysis of a microarray, next-generation sequencing, or deep sequencing data set. PLOS requires that authors comply with field-specific standards for preparation, recording, and deposition of data in repositories appropriate to their field.”

Sequence Read Archive (SRA) NCBI accession number added to revised cover letter.

4. “Please include captions for your Supporting Information files at the end of your manuscript, and update any in-text citations to match accordingly."

Captions for supplemental figures are included at the end of manuscript per the Reviewer’s request.

5. "Also, we need clarification if figures 1 and 2 were taking at the same coordinates (X,Y,Z) because the size looks significantly different.”

Yes, Figures 1 and 2 were taken at close proximal anatomical coordinates to obtain analysis of TH+ neurons, microglia and astocytes within the substantia nigra. The immunohistochemical images in Figure 1 provide a slightly different perspective than the immunofluorescence images in Figure 2 but every effort was made to include sections from the same relative anatomical plane through the basal midbrain.

6. Reviewer 2 comment: “These results add a new information that exposure to elevated levels of Mn during juvenile development could sensitize glial cells to more severe neuro-immune responses to influenza infection later in life through persistent epigenetic changes. In general, the study analyses are performed to a high technical standard and are described in sufficient detail; conclusions are presented in an appropriate fashion and are supported by the data.”

We thank the Reviewer for this critique and agree that the finding of neuro-immune priming of microglia by early life exposures to manganese that increases astrocyte reactivity upon challenge with H1N1 is an important finding in the field, especially considering the current global pandemic in which prior exposure to endogenous or exogenous toxins increases virulence. We suggest in the conclusions that this two-hit interaction between environmental toxins and infectious viruses may be important to understanding the etiology of various neurodegenerative diseases.
